# Effectiveness of late and very late antivenom administration on recovery from snakebite-induced coagulopathy in French Guiana: a population-based study

**DOI:** 10.1016/j.lana.2025.100994

**Published:** 2025-01-18

**Authors:** Jean Marc Pujo, Stephanie Houcke, Guy Roger Lontsi Ngoulla, Vivian Laurent, Boubacar Signaté, Rémi Mutricy, Alexis Frémery, Flaubert Nkontcho, Ibtissem Ben Amara, José María Gutiérrez, Dabor Resiere, Hatem Kallel

**Affiliations:** a Emergency Department, Cayenne General Hospital, Cayenne, French Guiana; b Tropical Biome and Immunopathology CNRS UMR-9017, Inserm U 1019, Université de Guyane, Cayenne, French Guiana; c Intensive Care Unit, Cayenne General Hospital, Cayenne, French Guiana; d Pharmacy Department, Cayenne General Hospital, Cayenne, French Guiana; e Amazin PopHealth, Département de Recherche et d’Innovation en Santé Publique (DRISP), Inserm Centre d’Investigation Clinique (CIC 1424), Cayenne Hospital Centre Andrée Rosemon, Cayenne, French Guiana; f Instituto Clodomiro Picado, Facultad de Microbiología, Universidad de Costa Rica, San José, Costa Rica; g Intensive Care Unit, Martinique University Hospital, Fort de France, Martinique

**Keywords:** Snakebite envenoming, Clotting time, French Guiana, *Bothrops atrox*, Antivipmyn Tri®

## Abstract

**Background:**

Snakebite (SB) envenoming is an acute emergency requiring early care delivery. However, sometimes, patients can take several hours before receiving antivenom (AV). We conducted this study to assess the effectiveness of antivenom in the recovery of clotting parameters in patients consulting tardily after SB envenoming in French Guiana. The primary endpoint of our study was to investigate the time needed from SB to recovery from SB-induced coagulopathy. The secondary endpoint was to investigate the time needed from AV administration to recovery from SB-induced coagulopathy in patients receiving AV (late or very late administration).

**Methods:**

This prospective observational study was conducted in the Intensive Care Unit (ICU) of Cayenne General Hospital between January 2016 and September 2023. We included all patients hospitalized for SB envenoming who either did not receive AV or received it more than 6 h after SB. We excluded patients who received antivenom in less than 6 h from the SB and those who received incomplete AV doses.

**Findings:**

We included 58 patients in the No AV group, 51 in the late AV group (6 h ≤ AV < 12 h), and 50 in the very late AV group (AV≥12 h). The median age of patients was 42 years (IQR: 29–53), 65.4% were male and 34.6% were female (104 and 55 out of 159 patients) without difference regarding the demographic parameters between groups. Data regarding ethnicity was not available. The median time from SB to AV was 8.5 h (IQR: 6.9–10) in the late AV group and 21.1 h (IQR: 16.7–27.4) in the very late AV group (p < 0.001). The time from SB to normal clotting parameters was shorter in patients receiving late AV than in those receiving very late AV and those not receiving AV. No differences were observed in the time from SB and recovery of fibrinogen and activated partial thromboplastin time (aPTT) between very late AV and no AV. However, the International Normalized Ratio (INR) recovery was shorter in the very late AV group than in the no AV group. On the other hand, the time from AV to normal fibrinogen was shorter in patients receiving very late AV than in patients receiving late AV (Log-Rank = 0.020). Meanwhile, the time from AV to normal INR or normal aPTT was similar in patients receiving very late AV compared to patients receiving late AV (Log-Rank = 0.722 and 0.740, respectively).

**Interpretation:**

Late AV administration effectively reverses coagulopathic manifestations after SB envenoming. However, very late AV administration did not improve the correction of some clotting parameters when compared to patients not receiving AV. Our findings could be explained by the combination of venom toxicokinetics and the kinetics of the synthesis of clotting factors.

**Funding:**

No funding.

**Research in context**

**Evidence before this study**

To identify the effect of time to antivenom (AV) on the outcome of snakebite envenomed victims, we searched the evidence available in PubMed for published works that evaluate the effect of time to AV administration on recovery of venom-induced coagulopathy and evaluated all human-focused publications including all genders, all age groups, and all regions of the world. We combined terms such as ((envenoming) OR (envenomation) OR (antivenom) OR (coagulation) OR (recovery) OR (early) OR (late)) AND (snakebite)). From these findings, we extracted the effect of early and late AV administration on the outcome of snakebite victims. There is widespread agreement that snakebite envenoming is a critical medical emergency that demands prompt intervention. Extensive literature exists estimating the superiority of early (<6 h) vs. late (≥6 h) administration of AV. In this context, scientists and the World Health Organization consider the early use of AV within the first 6 h of envenoming as the key to its effectiveness in preventing complications. However, there is no study evaluating the effectiveness of very late AV administration and at which point AV is no longer effective in reversing coagulopathy. Toxicokinetic investigations have revealed the persistence of certain snake venoms in the bloodstream beyond 24 h following the bite, with anecdotal reports indicating the potential efficacy of very late AV administration in reversing venom toxicity. Notably, no comparative study has evaluated the effectiveness of very late vs. late AV treatment or symptomatic management without AV.

**Added value of this study**

To our knowledge, this is the first study to introduce the concept of very late AV administration and to compare outcomes of patients receiving late and very late AV and those managed symptomatically without AV administration. Our results support the hypothesis that in the case of venom toxins having a short half-life, at a certain time after envenoming, there is no circulating venom, and the efficacy of AV administration is limited. Also, our results underscore the need to address unanswered questions regarding venom toxicokinetics according to snake species and locations.

**Implications of all the available evidence**

Our findings imply that AV might not be effective when administered very late in the course of envenoming. In addition, they support the idea that a broad strategy for the early use of AV should be paired with a conservative approach in scenarios where AVs are no longer effective. Further research focusing on the toxicokinetics of snake venoms and the pharmacokinetics of AV is required for a nuanced understanding of the natural progression of snakebite envenoming, contingent upon the snake species responsible and the timing of AV administration.

## Introduction

The World Health Organization (WHO) describes snakebite (SB) envenoming as an acute emergency requiring early care delivery.^1^ The WHO’s goal to halve the global burden of SB envenoming by 2030 can only be achieved by optimal and rapid access to safe and effective antivenom (AV) in the most affected regions. Indeed, the early use of AV is the key to its effectiveness in preventing complications.^2^, ^3^, ^4^ On the contrary, AV administration delays are associated with poor outcomes.^5^^,^^6^

On average, 90 snakebites are recorded yearly in French Guiana (FG),^7^, ^8^, ^9^, ^10^ and *Bothrops atrox* is the most involved snake.^8^^,^^11^ The AV used is Antivipmyn Tri®, deemed effective against the venom of most snakes encountered in the region.^12^ However, AV in FG is only available in the three main hospitals on the coast. Patients living in remote areas need to be transferred to the nearest hospital to benefit from appropriate care. AV administration delay can take up to 24 h in some cases^4^ and raises the question of the effectiveness of late or very late treatment. Indeed, toxicokinetic studies indicate that snake venom can be detected in the bloodstream more than 24 h after the bite.^3^^,^^13^ Furthermore, some case reports suggest that very late AV administration can effectively reverse the toxic syndrome.^14^, ^15^, ^16^, ^17^

In Latin America, early use of AV within the first 6 h of envenoming has been associated with better outcomes.^4^^,^^18^, ^19^, ^20^ In French Guiana, sixteen percent of the population (46,624/288,739 in 2024) lives in remote areas where a medical evacuation can last 24 h to reach the hospital and receive AV.^4^^,^^8^^,^^21^ However, no study compared the effectiveness of a very late AV treatment to symptomatic management without AV. We conducted this study to investigate the impact of late (6 h ≤ AV < 12 h) and very late AV (AV ≥ 12 h) administration, as compared to patients receiving no AV on the time lapse to recovery from SB induced coagulopathy in envenomed patients consulting belatedly in French Guiana.

## Methods

### Study design

This prospective observational study was conducted in the Cayenne General Hospital between 1 January 2016 and 30 September 2023.

### Participants

We included all patients hospitalized for SB envenoming, regardless of the grade of envenoming, who either did not receive AV or received it 6 or more hours after the bite. A group of patients did not receive AV because it was not available when these cases occurred. We excluded patients who received AV in less than 6 h from SB, as these patients were already investigated by our group and showed a shorter time to normalize clotting parameters.^4^ We also excluded patients who received less than six vials of AV which is the recommended dose according to the AV manufacturer and to the local protocol.^8^ AV administration and blood tests were performed according to the regional protocol.^8^

### Procedures

We prospectively collected epidemiological, clinical, and biological data at admission and during the hospital stay and recorded the adverse reactions to AV.

The study design and the regional AV protocol were previously described.^4^^,^^8^ The AV used is Antivipmyn Tri®, manufactured and marketed since 2008 by Instituto Bioclon, Mexico (Registry N 58583 SSA IV) and introduced in our hospital in 2017. Antivipmyn Tri® is a freeze-dried F(ab’)_2_ polyvalent AV prepared by immunizing horses with venoms of *Bothrops asper*, *Crotalus durissus terrificus*, and *Lachesis muta*. According to the manufacturer, one vial contains F(ab’)_2_ fragments neutralizing 780 LD_50_ (30 mg) of *B. asper* venom, 220 LD_50_ (15 mg) of *C. durissus* venom, and 200 LD_50_ (15 mg) of *L. muta* venom. The protocol of AV use was already described (6 vials, regardless of the grade of envenoming), and its efficacy and safety have been previously studied.^4^^,^^22^

The grade of envenoming was evaluated at patient admission to the medical service and hospital discharge and included three severity levels (Table 1).^9^ Worsening skin lesions refer to expanding local oedema, necrosis, or blisters over 24 h of surveillance.^4^ Expanding cutaneous oedema refers to the enlargement of the oedema zone by more than 5 cm in size. Expanding necrosis refers to the enlargement of the necrosis zone by more than 5 cm in size. Expanding blisters refers to the development of new blisters. Expanding skin manifestation is tracked by drawing a line around the lesion area with a marker and checking whether the lesion extends past the line after 24 h of medical observation.

**Table 1:** The grading system for classifying the severity of envenoming.

	Grade		
	I	II	III
Coagulation disorder^a^	**+**	**+**	**+**
Local signs			
Pain	**+**	**+**	**+**
Swelling	Not exceeding elbow or knee	Exceeding elbow or knee	Beyond the root of the limb
Blister	–	+	+
Necrosis	–	–	+
Local bleeding	–	+	+
Systemic bleeding	–	–	+
Systemic manifestations (Hypotension, Kidney Injury, Coma, Respiratory failure)	–	–	Organ failure

a Based on results of the 20-min whole blood clotting test and laboratory analysis.

Body Mass Index (BMI) was calculated using weight in kilograms divided by the square of height in meters. Thrombocytopenia was defined by a platelet count <150 G/L. Fibrinogen was measured by chronometric determination (Closs method) with a detection threshold of 0.35 g/L. Detectable fibrinogen was defined as a fibrinogen concentration higher than the threshold of detection using the dosage technique. Defibrinogenation was defined by a fibrinogen level <1 g/L (normal value: 2–4 g/L). Rhabdomyolysis was defined by a creatine kinase (CK) activity >500 IU/L (normal value: 39–308 IU/L). Coagulation disorders were defined by: International Normalized Ratio >2 (normal value: 0.8–1.2), Activated partial thromboplastin time >1.5, and Prothrombin time <60%. Renal impairment was defined according to the Kidney Disease Improving Global Outcomes (KDIGO) definition.^23^ The time to AV (the time course between the SB and the administration of AV) was classified as “late” (between 6 and 12 h) and “very late” (≥12 h).

The AV used is authorised “compassionately” by the French Agency for Drug Safety (code product: 3400893189627). The hospital’s institutional review board (Direction Qualité du Centre Hospitalier de Cayenne) and the Cayenne Hospital ethics committee (Comité d’éthique du Centre Hospitalier de Cayenne) approved the protocol of AV administration and blood test dosages (Ref: UF3700/17’, version “b”). Formal verbal consent was obtained from all patients or parents/guardians (when patients are <18 years old or unable to consent) and was reported in the patient’s medical file. In addition, at admission to our hospital, an information booklet was distributed to all patients or their relatives stating that their data may be used for research purposes and that they can oppose this use. The database has been registered at the “Commission Nationale de l’Informatique et des Libertés” (registration n 2217025v0) in compliance with French law on electronic data sources.

### Endpoints

The primary endpoint of our study was to investigate the time needed from SB to recovery from SB induced coagulopathy. The secondary endpoint was to investigate the time needed from AV administration to recovery from SB-induced coagulopathy in patients receiving AV (late or very late administration).

Resolved coagulopathy was defined as fibrinogen level >1 g/L, International Normalized Ratio <2, and Activated partial thromboplastin time <1.5. Coagulation disorders were monitored at admission and every 6 h thereafter until recovery.

### Statistical analysis

We created a data file with the patient’s information and performed a descriptive analysis using Excel (2007) and IBM SPSS Statistics for Windows, version 24 (IBM Corp., Armonk, NY, USA). Results are reported as the median and inter-quartile range (IQR) or numbers with percentages. We used the Chi-square or the Fisher exact test to compare qualitative variables. We used the Kruskal-Wallis test to compare quantitative variables. We used the Kaplan–Meier analysis and the Log-Rank test to compare the time needed to achieve normal coagulation parameters between groups. The type I error (alpha) was adjusted for the number of pairwise comparisons, and a value ≤ 0.016 was considered for statistical significance. Log-Rank for pairwise comparison was considered significant when ≤0.05.

### Role of the funding source

There was no funding source for this study.

## Results

**Fig. 1:**
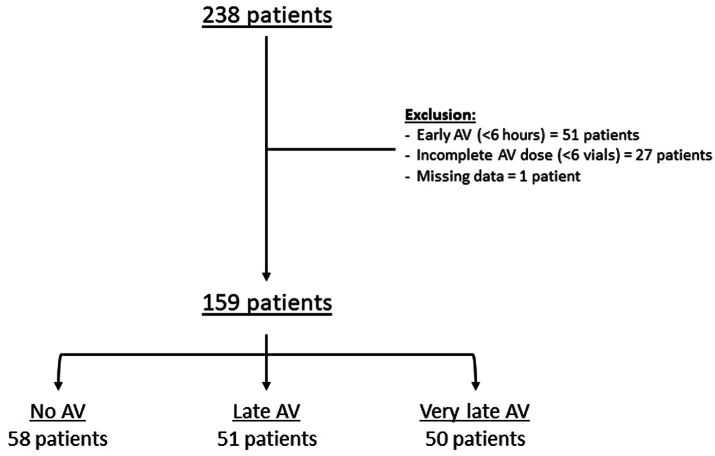
**Flow chart of the study.** AV: Antivenom, Late AV: 6 h ≤ AV < 12 h, Very late AV: AV ≥ 12 h.

During the study period, 238 patients were admitted to Cayenne Hospital with a diagnosis of SB envenoming, and 159 of them (66.8%) were included in our study. There were 58 in the No AV group, 51 in the late AV group, and 50 in the very late AV group (Fig. 1).

The median age of patients was 42 years (IQR: 29–53), 65.4% were male and 34.6% were female. There was no difference regarding the demographic parameters between groups. The responsible snake was identified in 67/159 cases (42.1%). It was *B. atrox* in 66 cases and *Bothrops bilineatus* in one case. The median time from SB to hospitalization was 18.3 h (IQR: 5.1–31.5) in the No AV group, 5.1 h (IQR: 2.1–7.3) in the late AV group, and 19 h (IQR: 14.9–26.1) in the very late AV group. The median time from SB to AV therapy was 8.5 h (IQR: 6.9–10) in the late AV group and 21.1 h (IQR: 16.7–27.4) in the very late AV group (p < 0.001). In the No AV group, the main clinical symptoms were oedema (56 out of 58 patients, 96.6%), pain (55 out of 58 patients, 94.8%), blisters (17 out of 58 patients, 29.3%), and skin necrosis (9 out of 58 patients, 15.5%). In the late AV group, there were fewer blisters (p = 0.011) and less extensive oedema, as compared to the No AV group (p = 0.018). In the very late AV group, clinical manifestations were statistically similar to those observed in the late AV group. Still, there was more oedema when compared with the late AV group (p = 0.043). Table 2 summarizes the epidemiological and clinical parameters on admission of the included patients.

**Table 2:** Baseline characteristics and clinical progression during hospitalisation.

Parameter	Total		No antivenom		Late antivenom		Very late antivenom		p
	Nb	Result	Nb	Result	Nb	Result	Nb	Result	
Age (years)	159	42 (29–53)	58	46 (28–51)	51	37 (24–57)	50	45 (31–54)	0.541
Male	159	104 (65.4%)	58	41 (70.7%)	51	29 (56.9%)	50	34 (68%)	0.285
Female	159	55 (34.6%)	58	17 (29.3%)	51	22 (43.1%)	50	16 (32%)	0.285
BMI (kg/m^2^)	97	23.8 (21.2–27.2)	49	23.8 (21.9–27.2)	23	23.1 (20.7–26.4)	25	23.9 (21.2–27.3)	0.613
Remote health centre	159	103 (64.8%)	58	41 (70.7%)	51	22 (43.1%)	50	40 (80%)	0.000
Past medical history	159	35 (22%)	58	13 (22.4%)	51	14 (27.5%)	50	8 (16%)	0.380
Arterial hypertension	159	7 (4.4%)	58	2 (3.4%)	51	3 (5.9%)	50	2 (4%)	0.815
Diabetes	159	5 (3.1%)	58	1 (1.7%)	51	3 (5.9%)	50	1 (2%)	0.396
Alcohol abuse	159	7 (4.4%)	58	4 (6.9%)	51	1 (2%)	50	2 (4%)	0.450
Time course									
Time from SB to hospitalization (hours)	159	10.1 (3.8–22.3)	58	18.3 (5.1–31.5)	51	5.1 (2.7–7.3)	50	19 (14.9–26.1)	0.000
Time from SB to hospitalization <24 h	159	123 (77.4%)	58	39 (67.2%)	51	49 (96.1%)	50	35 (70%)	0.001
Time from SB to hospitalization <48 h	159	144 (90.6%)	58	46 (79.3%)	51	51 (100%)	50	47 (94%)	0.001
Length of hospital stay, days	159	10 (6–14)	58	10 (6–19)	51	8 (5–11)	50	10 (6–15)	0.106
Clinical parameters									
Pain	159	155 (97.5%)	58	55 (94.8%)	51	50 (98%)	50	50 (100%)	0.221
Edema	159	153 (96.2%)	58	56 (96.6%)	51	47 (92.2%)	50	50 (100%)	0.116
Number of member segments with edema	159	1 (1–2)	58	2 (1–2)	51	1 (1–2)	50	2 (1–2)	0.004
Local haemorrhage	159	19 (11.9%)	58	6 (10.3%)	51	7 (13.7%)	50	6 (12%)	0.863
Necrosis	159	28 (17.6%)	58	9 (15.5%)	51	6 (11.8%)	50	13 (26%)	0.149
Blisters	159	30 (18.9%)	58	17 (29.3%)	51	5 (9.8%)	50	8 (16%)	0.028
Shock	159	3 (1.9%)	58	2 (3.4%)	51	0 (0%)	50	1 (2%)	0.417
Grade of envenoming									
Grade I	159	69 (43.4%)	58	22 (37.9%)	51	31 (60.8%)	50	16 (32%)	0.008
Grade II	159	46 (28.9%)	58	17 (29.3%)	51	12 (23.5%)	50	17 (34%)	0.508
Grade III	159	44 (27.7%)	58	19 (32.8%)	51	8 (15.7%)	50	17 (34%)	0.067
Grade progression during hospitalization	159	12 (7.5%)	58	3 (5.2%)	51	7 (13.7%)	50	2 (4%)	0.125
From grade I to grade II	153	9 (5.9%)	58	3 (5.2%)	47	5 (10.6%)	48	1 (2.1%)	0.199
From grade I to grade III	153	2 (1.3%)	58	0 (0%)	47	2 (4.3%)	48	0 (0%)	0.102
From grade II to grade III	153	1 (0.7%)	58	0 (0%)	47	0 (0%)	48	1 (2.1%)	0.333
Extensive lesions	159	41 (25.8%)	58	15 (25.9%)	51	13 (25.5%)	50	13 (26%)	0.998
Local edema	41	38 (92.7%)	15	15 (100%)	13	10 (76.9%)	13	13 (100%)	0.031
Blister	41	7 (17.1%)	15	2 (13.3%)	13	3 (23.1%)	13	2 (15.4%)	0.777
Necrosis	41	5 (12.2%)	15	0 (0%)	13	1 (7.7%)	13	4 (30.8%)	0.038
Kidney injury	159	30 (18.9%)	58	11 (19%)	51	8 (15.7%)	50	11 (22%)	0.720
Time from SB to kidney injury (days)	30	0 (0–2)	11	3 (1–4)	8	0 (0–1)	11	0 (0–1)	0.015
Time to normal kidney parameters (days)	28	8 (2–12)	11	11 (1–25)	7	3 (2–7)	10	8 (4–10)	0.513
Systemic haemorrhage	159	25 (15.7%)	58	13 (22.4%)	51	5 (9.8%)	50	7 (14%)	0.181
Time from SB to systemic haemorrhage (hours)	22	6 (1–22)	13	16 (5–24)	3	4 (3–7)	6	0 (0–3)	0.028

Results are expressed as numbers and percentages or median (IQR), Nb: the number of patients in whom the data was recorded, BMI: Body Mass Index, SB: Snakebite.

Symptomatic management was based on painkillers (100%) in all groups. Fluid infusion was used in 62.1% of cases (36 out of 58 patients) in the No AV group, 27.5% (14 out of 51 patients) in the late AV group (p < 0.001 compared to the No AV group), and 30% (15 out of 50 patients) in the very late AV group (p = 0.001 compared to the No AV group). Dialysis was used in 12.1% of cases (7 out of 58 patients) in the No AV group, 0% in the late AV group (p = 0.014 compared to the No AV group), and 2% (1 out of 50 patients) in the very late AV group (p = 0.066 compared to the No AV group). Surgery was required for 32.8% of cases (19 out of 58 patients) in the No AV group, 25.5% (13 out of 51 patients) in the late AV group, and 36% (18 out of 50 patients) in the very late AV group without significant differences (Table 3).

**Table 3:** Management of patients.

Parameter	Total		No antivenom		Late antivenom		Very late antivenom		p
	Nb	Result	Nb	Result	Nb	Result	Nb	Result	
Dialysis	159	8 (5%)	58	7 (12.1%)	51	0 (0%)	50	1 (2%)	0.008
Dialysis duration (days)	8	9 (4–15)	7	10 (4–20)	0	–	1	7 (7–7)	0.825
Fluid infusion	159	65 (40.9%)	58	36 (62.1%)	51	14 (27.5%)	50	15 (30%)	0.000
Fluid infusion volume (ml)	65	1500 (1000–2000)	36	1500 (1000–2000)	14	1000 (1000–1875)	15	1500 (1000–2250)	0.556
Catecholamines	159	3 (1.9%)	58	2 (3.4%)	51	0 (0%)	50	1 (2%)	0.417
Antibiotics at admission	159	61 (38.4%)	58	22 (37.9%)	51	14 (27.5%)	50	25 (50%)	0.066
Antivenom	159	101 (63.5%)	58	0 (0%)	51	51 (100%)	50	50 (100%)	-
Time from SB to AV (hours)	101	11.8 (8.5–21)	0	–	51	8.5 (6.9–10)	50	21.1 (16.7–27.4)	0.000
Early adverse reactions to AV	101	6 (5.9%)	0	–	51	2 (3.9%)	50	4 (8%)	0.386
Blood transfusion	159	15 (9.4%)	58	11 (19%)	51	1 (2%)	50	3 (6%)	0.006
Surgery	159	50 (31.4%)	58	19 (32.8%)	51	13 (25.5%)	50	18 (36%)	0.505
Time from admission to surgery (days)	50	5 (4–9)	19	5 (3–8)	13	6 (5–8)	18	5 (3–9)	0.485
Necrosectomy	50	23 (46%)	19	9 (47.4%)	13	3 (23.1%)	18	11 (61.1%)	0.110

Results are expressed as numbers and percentages or median (IQR), Nb: the number of patients in whom the data was recorded.

**Fig. 2:**
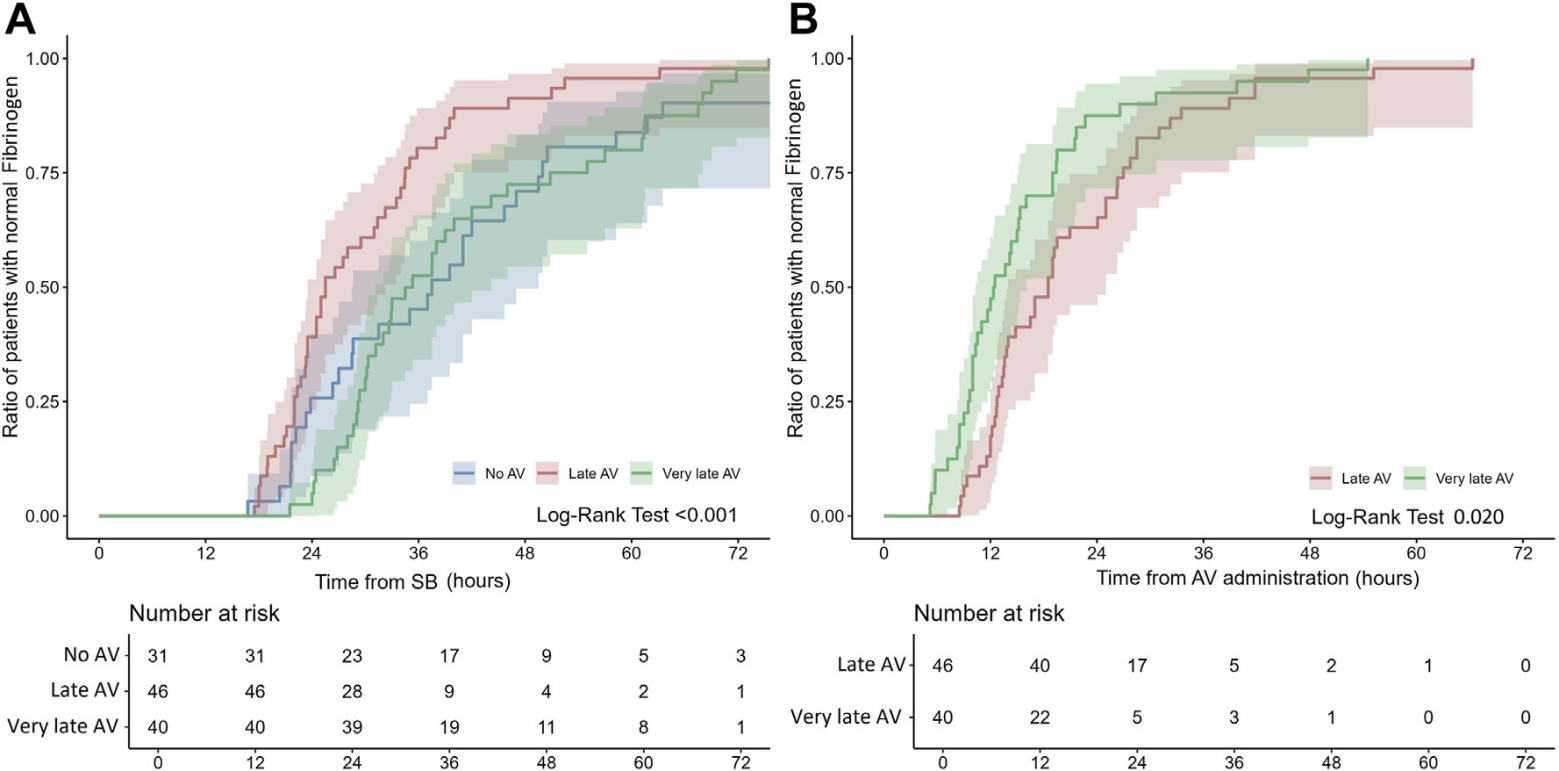
**The Kaplan–Meier analysis demonstrates (panel A) the time and 95% CI from the snake bite to the correction of fibrinogen concentration was shorter in patients receiving late AV (in red) than in those receiving very late AV (in green) and those who did not receive AV (in blue); (panel B) the time and 95% CI from the AV administration to the correction of fibrinogen concentration was shorter in patients receiving very late AV than in those receiving late AV**.

**Fig. 3:**
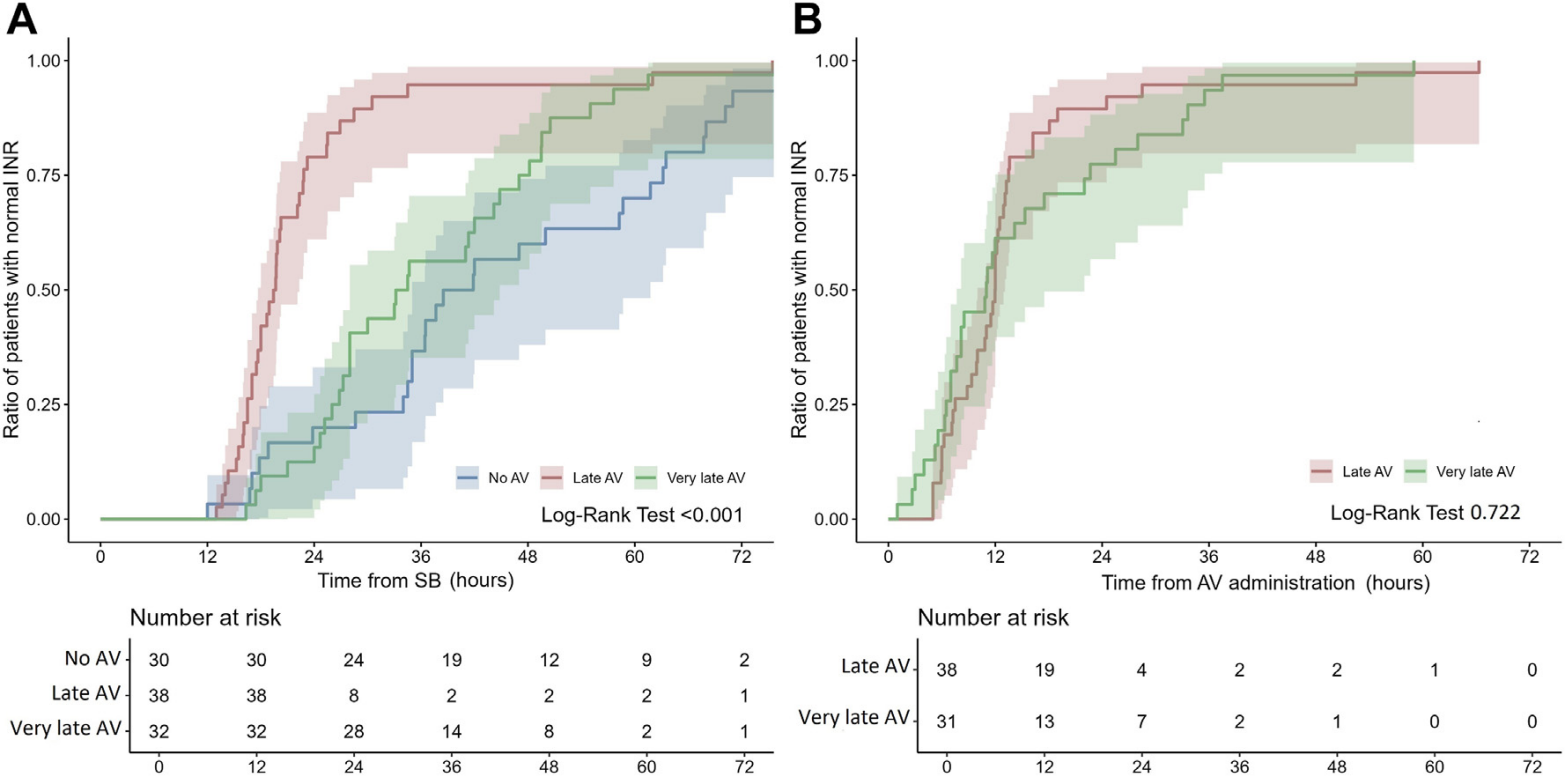
**The Kaplan–Meier analysis demonstrates (panel A) the time and 95% CI from the snake bite to a normal value of INR was shorter in patients receiving late AV (in red) than in those receiving very late AV (in green) and those who did not receive AV (in blue); (panel B) the time and 95% CI from the AV administration to a normal value of INR was similar in patients receiving late and very late AV (One patient received AV while the INR had already returned to normal)**.

**Fig. 4:**
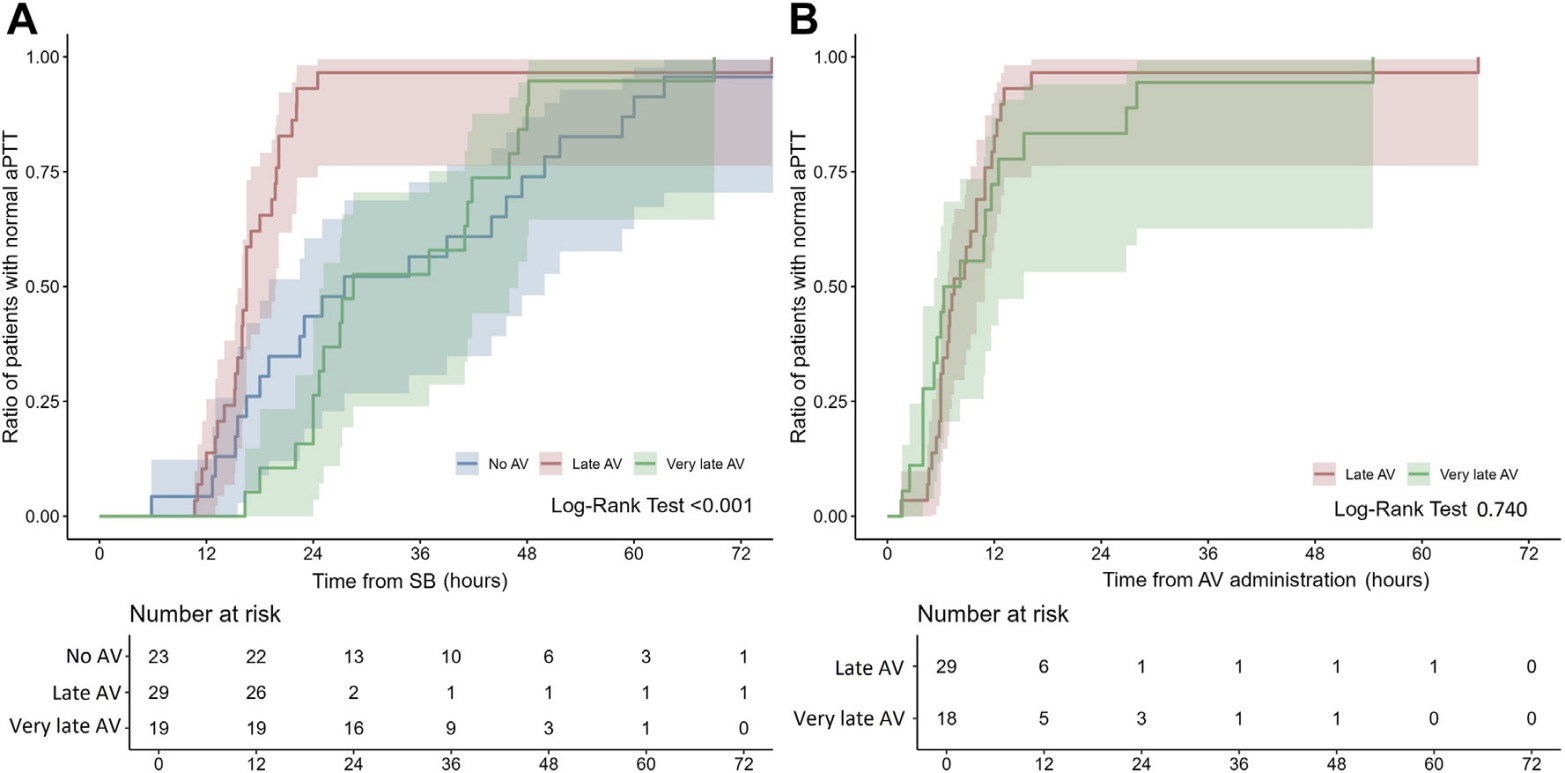
**The Kaplan–Meier analysis demonstrates (panel A) the time and 95% CI from the snake bite to a normal value of aPTT was shorter in patients receiving late AV (in red) than in those receiving very late AV (in green) and those who did not receive AV (in blue); (panel B) the time and 95% CI from the AV administration to a normal value of aPTT was similar in patients receiving late and very late AV (One patient received AV while the aPTT had already returned to normal)**.

Biological parameters at admission showed defibrinogenation in 63.8% of cases (37 out of 58 patients) in the No AV group, 94.1% (48 out of 51 patients) in the late AV group (p < 0.001 compared to the No AV group), and 86% (43 out of 50 patients) in the very late AV group (p = 0.009 compared to the No AV group, and p = 0.172 compared to the late AV group). Thrombocytopenia was observed in 39.7% of cases (23 out of 58 patients) in the No AV group, 19.6% (10 out of 51 patients) in the late AV group (p = 0.023 compared to the No AV group), and 26% (13 out of 50 patients) in the very late AV group (p = 0.133 compared to the No AV group, and p = 0.444 compared to the late AV group). INR was >2 in 55.2% of cases (32 out of 58 patients) in the No AV group, 78.4% (40 out of 51 patients) in the late AV group (p = 0.011 compared to the No AV group), and 72% (36 out of 50 patients) in the very late AV group (p = 0.071 compared to the No AV group, and p = 0.454 compared to the late AV group). aPTT was >1.5 in 46.6% of cases (27 out of 58 patients) in the No AV group, 56.9% (29 out of 51 patients) in the late AV group (p = 0.283 compared to the No AV group), and 38% (19 out of 50 patients) in the very late AV group (p = 0.058 compared to the No AV group). The time from SB to normal clotting parameters was shorter in patients receiving late AV than in those receiving very late AV and those not receiving AV. Kaplan–Meier analysis showed no significant difference in the time from SB to recover fibrinogen and aPTT between the very late AV and the no AV groups (Pairwise Log-Rank = 0.719 and 0.684, respectively). In contrast, the recovery of INR was slightly, but significantly, shorter in the very late AV group than in the no AV group (Pairwise Log- Rank = 0.028) (Fig. 2, Fig. 3 and 4). On the other hand, the time from AV to normal fibrinogen concentration was shorter in patients receiving very late AV than in patients receiving late AV (Fig. 2–Log-Rank = 0.020). Meanwhile, the time from AV to normal INR or normal aPTT was similar in patients receiving very late AV compared to patients receiving late AV (Figs. 3 and 4–Log-Rank = 0.722 and 0.740, respectively). Table 4 summarizes the biological parameters recorded at admission and during the hospital stay.

**Table 4:** Biologic abnormalities recorded at admission and during a hospital stay.

Parameter	Total		No antivenom		Late antivenom		Very late antivenom		p
	Nb	Result	Nb	Result	Nb	Result	Nb	Result	
Defibrinogenation	159	128 (80.5%)	58	37 (63.8%)	51	48 (94.1%)	50	43 (86%)	0.000
Time from SB to normal fibrinogen	117	32 (24.4–42)	31	37.5 (25.1–49.7)	46	25.5 (22.1–34.5)	40	34.9 (29.3–51.9)	0.001
Time from AV to normal fibrinogen	86	14.9 (11.1–23.7)	0	–	46	18.5 (12.8–26.8)	40	12.4 (9.6–19.1)	0.004
Fibrinogen dosage>0.35 g/L on admission	124	35 (28.2%)	36	9 (25%)	45	6 (13.3%)	43	20 (46.5%)	0.002
INR > 2	159	108 (67.9%)	58	32 (55.2%)	51	40 (78.4%)	50	36 (72%)	0.026
Time from SB to normal INR	100	28 (18.9–44.3)	30	40.2 (34.1–62.8)	38	19.5 (16.6–22.8)	32	33.8 (26.6–47.3)	0.000
Time from AV to normal INR	69	11.7 (7.1–16.2)	0	–	38	12 (7.8–13.4)	31	11 (6.8–22.3)	0.805
aPTT > 1.5	159	75 (47.2%)	58	27 (46.6%)	51	29 (56.9%)	50	19 (38%)	0.164
Time from SB to normal aPTT	71	22 (16.2–40)	23	27.5 (17.3–48.7)	29	16.5 (15.2–19.8)	19	28.5 (24.3–43.9)	0.000
Time from AV to normal aPTT	47	7.5 (5.7–11.7)	0	–	29	7.5 (6–10.9)	18	7.3 (4.3–12.3)	0.751
Thrombocytopenia	159	46 (28.9%)	58	23 (39.7%)	51	10 (19.6%)	50	13 (26%)	0.061
Time from SB to normal platelet count	38	110.3 (65.2–188.7)	20	174.8 (111.1–215.9)	8	66.5 (49.2–72.5)	10	59.6 (32.4–115.7)	0.002
Time from AV to normal platelet count	18	57.6 (12.4–75.6)	0	–	8	59.5 (40.2–63)	10	39.3 (10.7–100.9)	0.789
Platelets count (Giga/L)	45	110 (73–122)	23	89 (71–120)	10	120 (108–134)	12	113 (84–121)	0.247
Rhabdomyolysis	159	24 (15.1%)	58	10 (17.2%)	51	6 (11.8%)	50	8 (16%)	0.711
Time from SB to normal CK	11	44 (31.5–81.1)	5	44 (35–66)	2	20 (16–24)	4	95 (69.4–144.7)	0.072
Time from AV to normal CK	6	38.5 (15.5–79.3)	0	–	2	11.2 (7.7–14.6)	4	72.5 (47.9–107.9)	0.165
Haemolysis	159	42 (26.4%)	58	15 (25.9%)	51	13 (25.5%)	50	14 (28%)	0.953
Time from SB to resolved hemolysis	30	31.4 (23.6–70.1)	11	71 (48.3–125.6)	12	23.9 (19.3–26.4)	7	39.4 (25.5–71)	0.004
Positive schizocytes	28	5 (17.9%)	7	4 (57.1%)	10	1 (10%)	11	0 (0%)	0.006
Schizocytes (%)	5	1.3 (1.07–1.5)	4	1.19 (1.05–1.35)	1	4	0	–	–

Results are expressed as numbers and percentages or median (IQR), Nb: the number of patients in whom the data was recorded, Time courses from SB and AV are expressed in hours, INR: International Normalised Ratio, aPTT: adjusted Prothrombin Time, CK: Creatine Kinase, SB: Snakebite.

## Discussion

The timing in the administration of AV plays a key role in the outcome of SB envenoming. Our results show that patients receiving late AV, i.e., between 6 and 12 h after the bite, took a shorter time to restore clotting parameters than patients receiving very late AV, i.e., more than 12 h after the bite, and those not receiving AV. Also, there is a general trend that clotting parameters recovery is similar in the very late and the No AV groups. Accordingly, the benefit of AV decreases with the delay of administration and is largely absent in terms of correction of clotting parameters in the case of very late treatment in envenomings by *B. atrox* in French Guiana.

SB envenoming is a severe and disabling disease mainly affecting impoverished and disadvantaged communities with limited access to medical care. Furthermore, in some populations, the reluctance of SB victims to medical care, favouring traditional medicine, can be responsible for delays in appropriate treatment. In French Guiana, Houcke et al. reported that the median time from SB to AV was 9 h while it was 17 h in patients from remote areas.^4^ Several studies carried out in the Amazon region of Brazil, and several countries in sub-Saharan Africa described a prolonged delay in AV administration in many cases.^24^, ^25^, ^26^, ^27^, ^28^, ^29^ In contrast, in other settings such as Australia, Sri Lanka, and Costa Rica, AV was administered within the first hours after the bite.^3^^,^^30^^,^^31^

In French Guiana, one of the most challenging clinical decisions in SB management is whether to give AV to envenomed patients attending the hospital very late after the bite. To address this issue, snake venom toxicokinetic can provide important information about the time course of envenoming. It may explain how long AV remains an effective therapeutic intervention after the onset of envenoming. Studies on venom toxicokinetic have revealed a rapid distribution phase of toxins followed by a slow elimination phase.^13^ However, venoms having different toxin compositions differ in their distribution and elimination half-lives.^3^^,^^13^^,^^32^, ^33^, ^34^ In addition, different toxins in a single venom may have variable toxicokinetic profiles.^35^ A theoretical model predicted a rapid elimination from the circulation of procoagulant toxins of taipan (*Oxyuranus scutellatus*) venom.^36^ Moreover, toxicokinetic is impacted by the accumulation of venom in tissue depots, from which toxins are released, thus prolonging their distribution into the bloodstream. Therefore, the variable toxicokinetic profiles of venoms impact the dynamics of the envenoming process and the time interval when AV is effective, which may vary depending on the venom and the circumstances of envenoming.

**Fig. 5:**
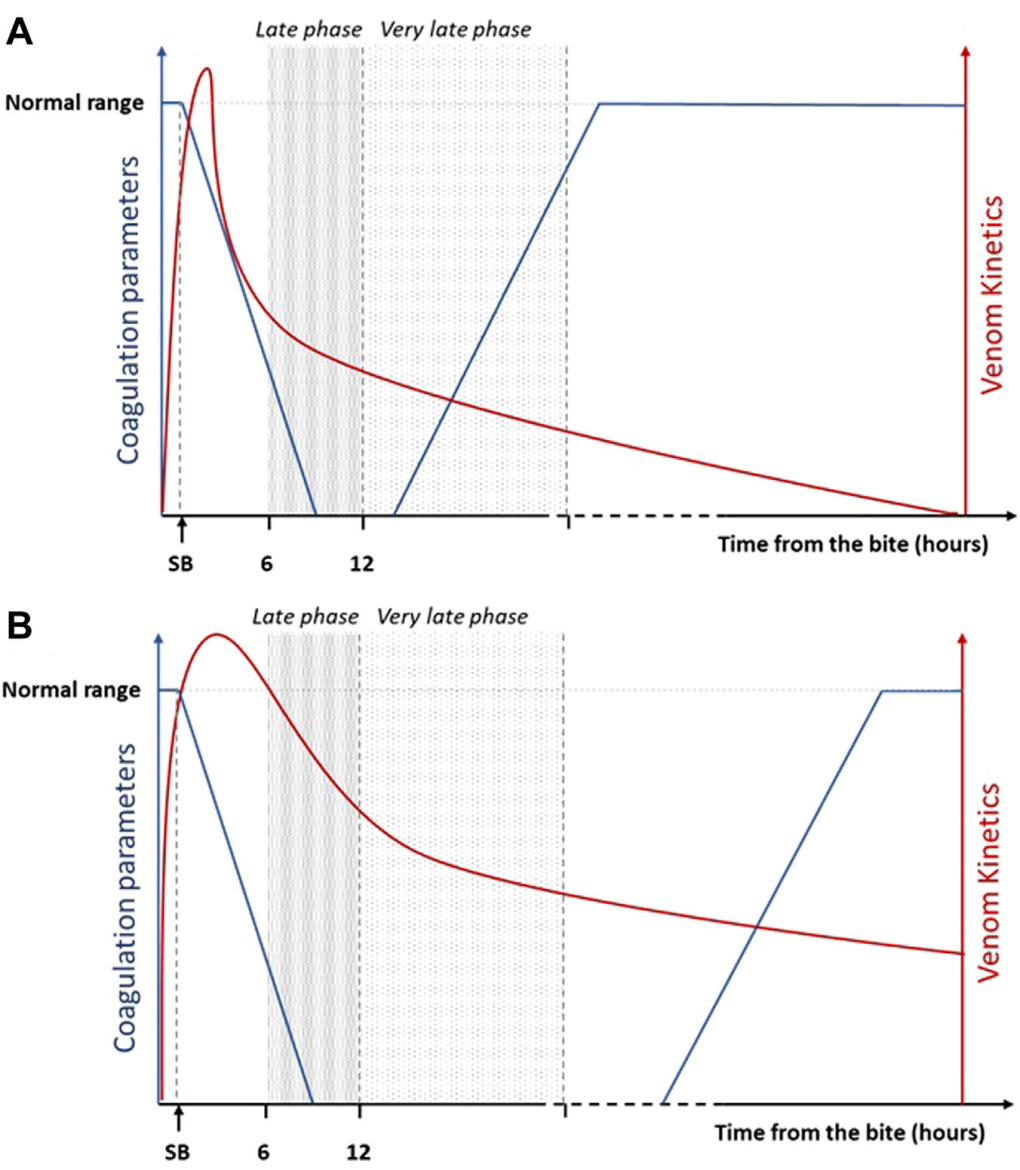
**Hypothetical scheme of the venom toxicokinetics, as related to consumption and recovery of clotting factors *vis-à-vis* late and very late phases of AV administration.** (A) Short half-life venom: Rapid elimination of the circulating venom and the natural reversion of coagulation factors explain the lack of effect of very late AV (B) Long half-life venom: Low elimination of the circulating venom and the late natural reversion of coagulation disorders explain the efficacy of very late AV in fostering the recovery of clotting parameters.

There is limited clinical information on the efficacy of late AV administration on the recovery from clotting disturbances in envenomings. For example, Boyer et al. described a persistent coagulopathy after pit viper envenoming after two weeks in North America,^37^ and Mion et al. described that, in *Echis* sp. envenoming, haemostasis remains severely affected until the 8th to 10th day of evolution in the absence of AV administration.^38^ Fig. 5 presents a hypothetical scheme comparing two scenarios differing in the elimination half-lives of coagulopathic venoms, in which venom elimination, consumption of clotting factors, and synthesis of clotting factors by the liver are considered. For example, two simultaneous processes determine the recovery of fibrinogen levels, i.e., AV’s neutralization of venom procoagulant enzymes and the liver’s replenishment of fibrinogen and other clotting factors. Thus, it is hypothesized that, in the case of very late AV (more than 12 h), an increased synthesis of fibrinogen has already started, the venom has been largely eliminated, and the recovery of clotting parameters is ongoing.^4^ A delayed AV administration would effectively speed up the recovery of clotting parameters in cases where procoagulant toxins remain in the bloodstream for prolonged periods. In the present study, AV proved effective in reversing coagulopathy when administered within the first 12 h after envenoming but did not modify the recovery of clotting parameters, as compared to cases in which no AV was given when provided after the 12th hour. These findings should not be extrapolated to envenomings by other snake species and AVs or different doses of the same AV. Moreover, the fact that very late administration of AV did not improve the correction of clotting disturbances does not imply that such late administration is unfavourable in other aspects of the envenoming syndrome, an issue that requires further studies. Some case reports have described a favourable outcome in envenomed patients receiving very late AV.^14^, ^15^, ^16^, ^17^

Our observations underscore the impact of the time of AV administration on the correction of coagulopathy, a relevant aspect of *B. atrox* envenoming. In South America, it is well established that early AV administration (<6 h post-bite) is associated with better outcomes.^4^^,^^24^ The delay in AV administration is directly linked to the availability of AV in different settings. In FG, AV is available only in the three main hospitals of the territory, and the first acute medical care is steered by the Regional Call Centre for Emergencies, SAMU 973. Although the one-trip access time to the nearest hospital can be about 10–20 min in urban cities (by car), it can go up to 100 min from isolated zones (by helicopter).^4^ In French Guiana, patients from remote sites must attend the local healthcare centre before being transferred to Cayenne Hospital. Sometimes, patients must go through the forest or rivers to reach the healthcare centre. In addition, roads exist only on the coastal side and the only means to medevac a patient from most remote areas remains by helicopter. Thus, AV administration is delayed in several cases. The delay in attending a healthcare facility and receiving appropriate treatment is a typical picture of inequitable access to healthcare in the Guianese population. Thus, the improvement in the management of SB envenoming in FG must involve the availability of AV and training programs for health staff in remote healthcare facilities to reduce the time lapse between SB and therapy.^39^

We are aware that our study may have some limitations. The time to recovery analysis was based on univariable analysis without adjusting for the grade of envenoming. However, the grade of envenoming primarily considers local and systemic manifestations, which do not necessarily correlate with the extent of coagulopathy observed. Furthermore, it is important to highlight that this is the first study assessing the effectiveness of very late AV administration on time to recovery from snakebite-induced coagulopathy in patients consulting belatedly after SB envenoming. Conclusions should be drawn cautiously, and similar investigations with larger samples should be conducted in endemic regions. Also, our study shed light on the need for toxicokinetic data of the venom to guide the optimal time to administer antivenom.

### Conclusion

Our results suggest that patients receiving late AV, i.e., between 6 h and 12 h after SB, had a shorter time to reach normal clotting parameters than patients who did not receive AV. However, very late AV, i.e., administered after 12 h, seems to be less effective in speeding the recovery of coagulation disorders after SB envenoming in French Guiana. More in-depth studies on the toxicokinetic of snake venom and the pharmacodynamics of AV are needed to better understand the natural history of SB envenomation based on the culprit snake and the AV used. Supplying remote rural healthcare facilities with AV can shorten the time course from SB to AV and improve the outcome of envenomed patients.

## Contributors

Every author named at the start of the Article contributed to the study. All authors had full access to all the data in the study and had final responsibility for the decision to submit for publication. HK, SH, and GRLN have accessed and verified the data.

JMP: conceptualisation, data curation, methodology, project administration, writing original draft.

SH: data curation, methodology, project administration, validation, writing review & editing.

GRLN: data curation, investigation, software.

VL: data curation, methodology, writing original draft.

BS: conceptualisation, data curation, writing original draft.

RM: data curation, software, validation.

AF: data curation, project administration, software.

FN: data curation, validation, visualisation, writing review & editing.

IBA: methodology, project administration, writing review & editing.

JMG: conceptualisation, supervision, validation, writing review & editing.

DR: conceptualisation, methodology, writing review & editing.

HK: conceptualisation, data curation, formal analysis, methodology, project administration, supervision, validation, writing original draft.

## Data sharing statement

The data that support the findings of this study are available from the corresponding author, HK, upon reasonable request.

## Declaration of interests

All authors declare no competing interests.

## Appendix A Supplementary data

Study Protocol
