# Supplementary material for: Effectiveness of late and very late antivenom administration on recovery from snakebite-induced coagulopathy in French Guiana: a population-based study
Source: Lancet Reg Health Am. 2025 Jan 18;42:100994. doi: 10.1016/j.lana.2025.100994 (PMC11787608; doi:10.1016/j.lana.2025.100994)
Supplement: Study Protocol [file mmc1.pdf]

| Titre : Protocole de prise en charge des envenimations par vipéridés en Guyane | | |
|---|---|---|
| Document organisationnel : ☐<br>Document opérationnel : ☒<br>Protocole de recherche : ☒ | **SECTEUR EMETTEUR :**<br>**Sce de Réanimation Polyvalente**<br>**Sce des Urgences SAMU** | Page :1/9<br>Date d'émission : 23/01/2016<br>Date première révision : 02/04/2017<br>Date dernière révision : 22/02/2024 |
| **Rédaction :**<br>Nom : Dr HOUCKE S.<br>Fonction : Médecin Réanimation | **Vérification :**<br>Nom : Dr DESCHAMPS C.<br>Fonction : Médecin Urgences | **Vérification :**<br>Nom : Dr François COISNE<br>Fonction : Comité d'éthique |
| **Approbation :**<br>Nom : Dr PASSARD S.<br>Fonction : Pharmacie | **Approbation :**<br>Nom : Pr PUJO JM.<br>Fonction : Chef de service Urgences | **Approbation :**<br>Nom : Pr KALLEL H.<br>Fonction : Chef de service Réanimation |
| **Ont collaboré à la rédaction : Equipe d'infectiologie du CHC et CHOG, équipe des urgences du CHOG, équipe de réanimation du CHOG** | | **Visa qualité : OK**<br>**Visa Comité d'éthique : OK** |
| **Services concernés par application :** URGENCES, REANIMATION | | |

## I- INVESTIGATEURS PRINCIPAUX

- Professeur Hatem KALLEL
- Professeur Jean Marc PUJO
- Dr Stéphanie HOUCKE

## II- OBJECTIF DU TRAVAIL

Monitorage des troubles de l'hémostase chez les patients victimes d'envenimation vipérine
Surveiller l'efficacité et les effets indésirables du traitement par Antivipmyn TRI®
Evaluation des délais d'administration de l'antivenin sur les délais de correction des troubles de l'hémostase

## III- POPULATION CONCERNEE

Tous les patients présentant une envenimation vipérine (morsure de serpent avec injection de venin).

## IV- TEXTE DE REFERENCE

Conclusions of the International symposium on the Epidemiology and the Management of Snakebites in French Guiana 2017.
Houcke et al. Characteristics of Snakebite-Related Infection in French Guiana. Toxins. 2022.24;14(2):89.

Houcke et al. Effect of the time to antivenom administration on recovery from snakebite envenoming-related coagulopathy in French Guiana. PLoS Negl Trop Dis. 2023.24;17(4):e0011242.

## V- DEFINITIONS

L'envenimation par vipéridés (Bothrops, Lachesis, Crotalus) associe un syndrome vipérin avec signes locaux (douleur, œdème, phlyctène et nécrose) et des troubles de l'hémostase (fibrinolyse aigue, coagulopathie, thrombopénie, hémolyse).

Le tableau clinique est habituellement peu symptomatique (50%), mais il peut évoluer jusqu'à la défaillance d'organes.

La classification clinique est essentielle au traitement et à la prise en charge.

## VI- DESCRIPTION

### A. Sur site :

- Rassurer le patient et le transporter le plus rapidement au CHC.
- Si transport médicalisé : 2 VVP et analgésie.
- Pas de garrot, pas d'aspi-venin, pas de scarification, pas de cautérisation
- Pas de corticoïdes, pas d'AINS, pas d'antibiotique.

### B. A l'arrivée à l'hôpital :

**<u>L'administration de l'antivenin est une URGENCE THERAPEUTIQUE.</u>**

- Pour les CDPS : appel du Centre 15.
- Pour les hôpitaux du GHT : appel systématique du réanimateur référent GHT : 0594397470 depuis l'extérieur/4570 en interne au CHC ou via le 15.

Elaboration d'une fiche de traçabilité par le réanimateur ou urgentiste référent.

**<u>Admission directe dans un des secteurs de soins critiques.</u>**

- Prise des constantes du patient.
- Antécédents du patient (prise d'anticoagulants, risque hémorragique, …).
- Données anamnestiques : date et heure de la morsure, lieu, circonstances, description du serpent et identification (photographie, spécimen).
- Evaluation des signes locaux : douleur, extension de l'œdème, saignement, phlyctène, nécrose (photographie des lésions pour le dossier médical).
- Evaluation des signes généraux : examen clinique complet.
- Evaluation des troubles de la coagulation en URGENCE
  - Test de coagulation à 20 minutes sur sang total
  - Bilan biologique

## 1. Prise en charge symptomatique :

- Analgésie selon prescription, pas d'AINS, pas de corticoïdes,
- Soins locaux : désinfecter le site de la morsure, délimiter l'œdème (marqueur) et mesurer le périmètre des membres concernés puis surélever le membre envenimé,
- Patient au repos strict au lit, à jeun (eau pour confort), pas de geste à risque hémorragique (brossage de dent, rasage, ponction artérielle, etc).

## 2. Bilan d'admission :

### *a. Le bilan biologique*

Le bilan biologique est protocolisé et comprend :

- **3 tubes violet** (EDTA) : NFS, schizocytes, groupe sanguin et RAI,
- **1 tube bleu** (Citrate) : TP, TCA, fibrinogène, D-dimères,
- **1 tube jaune** (sec) : troponine,
- **1 tube vert** (hépariné) : Sodium, Potassium, Chlore, Réserve alcaline, Urée, Créatinine, Bilan hépatique, LDH et CPK, haptoglobine,
- **1 tube gris** : lactate,
- et **1 tube rouge** (ne le remplir qu'à moitié, à garder dans l'unité).

PAS DE PONCTION ARTERIELLE +++ sauf prescription médicale.

Prélever ce bilan **toutes les 6 heures** tant que le bilan d'hémostase du patient n'est pas corrigé : taux de fibrinogène > 1g/l.

### *b. Le tube ROUGE*

Le tube ROUGE permet de détecter les troubles de la coagulation au lit du malade.
**A GARDER DANS L'UNITE pour vérification de la coagulabilité au bout de 20 minutes après le prélèvement.**
**Après prélèvement, déposer le tube sur le porte-tube en position verticale sans le toucher pendant 20 minutes jusqu'à sa lecture. Noter le résultat sur la feuille de surveillance.**
Jeter le tube après lecture à 20 min.

Si pas de caillot visible = administration de l'antivenin.

### *c. Autres examens*

- ECG,
- Tétanos Quick Test,
- CRP, PCT/48h,
- 2 paires d'hémocultures si T > 38,3°C.

***En fonction de l'examen clinique et du résultat du test de coagulation : gradation de l'envenimation selon la classification :***

| | | GRADE | | |
|---|---|---|---|---|
| | | I | II | III |
| Signes locaux | Douleur | + | + | + |
| | Œdème | Ne dépassant pas le genou ou le coude | Dépassant le genou ou le coude | Au-delà de la racine du membre |
| | Phlyctène | | + | + |
| | Nécrose | | | + |
| Trouble de la coagulation | | + | + | + |
| Manifestations systémiques | | | | Défaillance d'organe |

## 3. Prise en charge spécifique : l'antivenin

Immunothérapie = antivenin = Antivipmyn TRI® ou BOTHROFAV®

**Indication :**
L'administration de l'antivenin est réalisée sur prescription médicale en cas d'absence de coagulation sur le tube rouge et/ou du bilan biologique.

Préparer dans la chambre du patient une seringue d'Adrénaline 1mg/10ml (0,1mg/ml) avant toute administration de l'antivenin.

**PROCEDURE :**
1. Réalisation de l'ordonnance par le médecin. La prescription se fait en urgence sur une ordonnance vierge. La régularisation sur le site de l'ANSM (ATU) se fait dans un second temps.
La quantité d'antivenin à administrer est de 6 flacons quel que soit la gradation de l'envenimation

2. Appel du pharmacien par l'IDE en charge du patient pour sa délivrance.

3. Dispensation de l'antivenin par le pharmacien.

4. Préparation par l'IDE : reconstitution et dilution de chaque flacon à part (cf. Tableau).

5. Rester dans la chambre du patient pendant toute la durée de l'administration de l'antivenin et prendre les paramètres vitaux toutes les 5 minutes. Risque élevé de réaction anaphylactique.

6. Reporter les constantes sur la fiche de surveillance en annexe 1.

7. Si réaction anaphylactique (rash, prurit, hypotension, dyspnée), arrêter immédiatement l'administration de l'antivenin et appeler le médecin (annexe 2).

8. Surveillance clinique et biologique avec répétition du bilan biologique toutes les 6 heures.

## 4. CAS PARTICULIERS :

- Femme enceinte : pas de CI pour Antivipmyn TRI® / BOTHROFAV®.
- Enfant : même posologie que l'adulte.
- Insuffisance rénale aigue ou chronique : pas de modification de la posologie.

## 5. Orientation du patient selon la gradation :

- A H6 : test de coagulation et bilan biologique.

Si le test de coagulation est positif (caillot) à H6 et bilan biologique normal, refaire un bilan biologique à H12 et surveillance aux urgences.

- A H12 : si le bilan de coagulation est normal :
  - Présence de signes locaux : hospitalisation en service conventionnel.
  - Pas de signes locaux : retour à domicile avec conseils.

## 6. Prise en charge anti infectieuse :

**Pas d'antibioprophylaxie. Pas d'Amoxicilline - acide clavulanique.**

**Hémocultures et prélèvements microbiologiques avant toute antibiothérapie.**

- **Fasciite nécrosante** : avis chirurgical en urgence, prélèvements microbiologiques per opératoire, PIPERACILLINE-TAZOBACTAM
- **Sepsis** : PIPERACILLINE-TAZOBACTAM
- **Choc septique** : PIPERACILLINE-TAZOBACTAM et AMIKACINE

La durée de l'antibiothérapie sera à discuter au cas par cas.

Dans tous les autres cas (fièvre, signes locaux, phlyctènes...) : il n'y a pas d'urgence à débuter une antibiothérapie, qui devra être discutée au cas par cas avec **l'équipe de réanimation du CHC** et introduite après hémocultures/prélèvements microbiologiques.

Les molécules qui doivent être privilégiées pour l'antibiothérapie préemptive sont les suivantes :
- **LEVOFLOXACINE PO 3 à 5 jours**
- **CEFOTAXIME IV 3 à 5 jours**
- **PIPERACILLINE-TAZOBACTAM**

L'antibiothérapie doit être réévaluée en fonction des prélèvements microbiologiques et de l'évolution du patient.

## VII- ANALYSE DE DONNEES

L'analyse de données est réalisée de façon périodique et à la demande de l'un des investigateurs pour répondre aux objectifs déjà fixés.

1- Pour l'étude descriptive, nous calculons la médiane, et l'étendue interquartiles (EIQ) des variables quantitatives et la fréquence des variables qualitatives.
2- Pour l'étude analytique, nous utilisons le test de Chi-deux pour comparer les variables qualitatives et le test de Mann et Whitney pour la comparaison de moyennes.
3- Pour déterminer la liaison entre deux variables quantitatives, nous réalisons une étude par régression linéaire avec calcul du coefficient de détermination (R2).
4- Pour déterminer le lien entre une variable quantitative dépendante et plusieurs variables quantitatives indépendantes, nous réalisons la régression linéaire multiple.
5- Les variables pertinentes associées à la variable d'intérêt dans l'analyse univariée sont introduites dans un modèle de régression logistique ascendant avec calcul de l'odd ratio (OR) et l'intervalle de confiance à 95 % [IC 95 %].
6- L'analyse du délai de correction des test de coagulation est réalisée en utilisant des analyses de survie avec calcul du « Log-Rank ».
6- Une valeur du risque de première espèce inférieure à 5% est considérée comme significative.

## VIII- ANNEXES

**Annexe 1 :** feuille de surveillance.

### Feuille de surveillance / administration antivenin

<u>Nom du patient:</u>

Etiquette patient

Date d'administration:

Heure de début de l'administration :

Heure de fin d'administration:

| | H0 | H 30 min | H 60 min | H 90 min | H 120 min | H 180 min | H 210 min | H 240 min | H 270 min | H 300min |
|---|---|---|---|---|---|---|---|---|---|---|
| TA | | | | | | | | | | |
| FC | | | | | | | | | | |
| FR | | | | | | | | | | |
| Sa02 | | | | | | | | | | |
| Signes cliniques | | | | | | | | | | |

**Annexe 2 :** prise en charge de l'anaphylaxie - recommandations 2016.

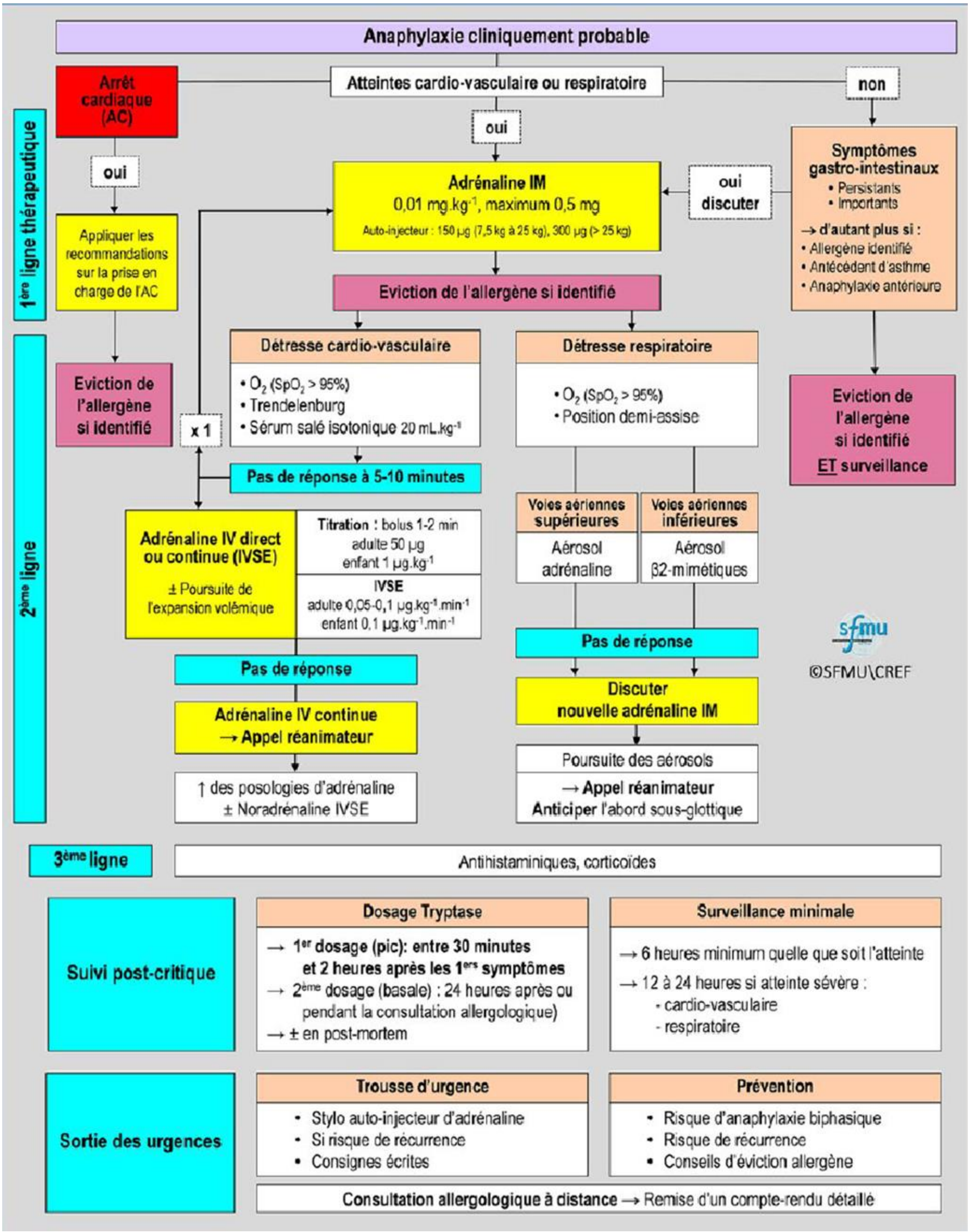

Document validé par :

Professeur Hatem KALLEL

Pr. KALLEL Hatem
Chef de Service de Réanimation
Centre Hospitalier de Cayenne
N° RPPS: 10100142388

Professeur Jean Marc PUJO
